# Novel hydrophobic catalysts to promote hydration at the water–oil interface

**DOI:** 10.1039/d1ra01188j

**Published:** 2021-05-20

**Authors:** Shuai Liu, Dahai Sun, Hui Tian

**Affiliations:** College of Chemistry and Chemical Engineering, Yantai University Yantai 264005 China tianhui@ytu.edu.cn +8618754591289

## Abstract

The limitation of the cyclohexene hydration reaction is that it is a three-phase immiscible reaction. We have described a strategy to overcome this interfacial mass transfer limitation by grafting an organosilane surfactant ((octyl)-trimethoxysilane (OTS)) onto the HZSM-5 zeolite surface. The characterization of the OTS-HZSM-5 zeolite was performed by FTIR, CA, BET, TPD, pyridine-IR, XPS, TGA and XRD techniques. The functionalization of the HZSM-5 zeolite could increase hydrophobicity without significantly reducing the density of acid sites. As a result, the OTS-HZSM-5 zeolite had high catalytic activity (20.87% conversion) compared with HZSM-5 (4.15% conversion) at 130 °C after 4 h. The high catalytic activity makes it a promising candidate for other acid-catalyzed two-phase reactions.

## Abstract 2

The limitation of the cyclohexene hydration reaction is that it is a three-phase immiscible reaction.

## Graphical abstract

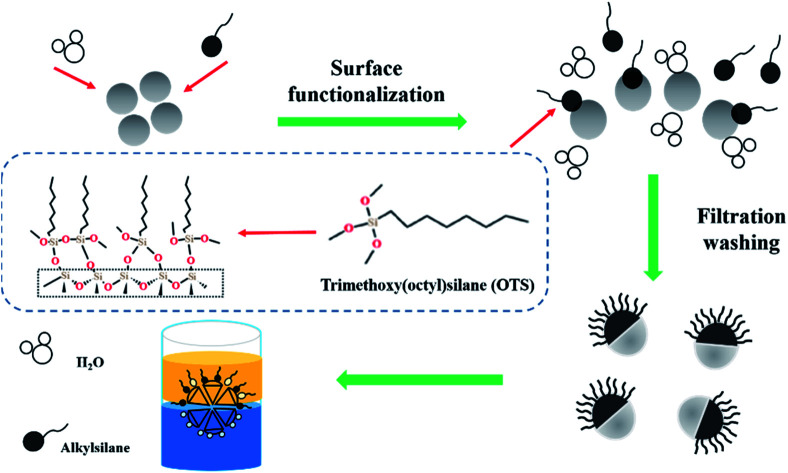

## 1. Introduction

Cyclohexanol is an important intermediate material for the production of adipic acid and caprolactam. Adipic acid is used as an important raw material for polymerization to produce nylon 66 and synthetic polyurethane, and caprolactam is used in the production of engineering plastics.^1–6^ Cyclohexanol is often used as a solvent for paints and varnishes in coatings, and as a matting agent in textiles.^7,8^ Therefore, cyclohexanol is an important chemical raw material.

Cyclohexene hydration is a heterogeneous immiscible reaction, in which the solid catalyst is at the bottom of the water phase, which cannot contact cyclohexene.^9–13^ In fact, numerous scholars have done research on the modification of ZSM-5. Spod *et al.*^14^ used HZSM-5 as a catalyst for cyclohexene hydration, in which the cyclohexene conversion could reach up to 13%. Nur *et al.*^15^ carried out the silylation of HZSM-5 and NaY with sulfanilic acid to prepare 1,2-epoxyoctane. They found that the active sites were mainly located on the inner surface of the zeolite. Therefore, they implemented the concept of the amphiphilic catalyst by partially covering it to improve conversion. A study by Shaodan Xu *et al.*^16^ showed that the hydrophobic zeolites were prepared by hexadecyltrimethoxysilane (C16). The contact angle of ZSM-5 was improved to 134° by functionalization. Zapata *et al.*^17^ recently obtained the hydrophobic HY zeolite by functionalization with organosilanes, which is more stable in hot water than the untreated zeolite. The hydrophilicity/hydrophobicity of the zeolite received increased attention due to its significant influence on the physical and chemical properties and application of zeolites.^18–23^

A method without reducing the acid site density is the silylation of the external surface using organosilanes, which could increase hydrophobicity.^24^ A remarkable characteristic of these material is their high affinity towards liquid–liquid interfaces, which stabilizes the emulsions of small droplet size. Hydrophobic materials could form oil-in-water and water-in-oil in a water–oil environment, which greatly increase the liquid–solid–liquid interface area and facilitate the separation of molecules from the reaction system.^25^

In this study, the silylation of the zeolite is shown to be significantly more active in cyclohexene hydration. To functionalize the silanol groups, the OTS-HZSM-5 zeolite was prepared by (octyl)-trimethoxysilane (OTS). Compared to other silylation reagents, the advantage of OTS is that it has three methoxy groups. We proposed the “triangle Si–O–Si” bond hypothesis as it could form a “triangular Si–O–Si” bond with the zeolite surface, which the three methoxy groups could combine with silanol groups to form a three-dimensional triangle. Simultaneously, the adjacent OTS was connected by “Si–O–Si” bonds to ensure the steady adsorption of the C8 hydrophobic chain. Since the catalyst was in the middle of the phase interface, the hydration system could form a Pickering emulsion by heating. The Pickering emulsion enhanced the interfacial mass transfer by increasing the contact surface between the two immiscible reactants.^26^ The OTS-HZSM-5 zeolite stabilized water/oil emulsion to solve the limitation in the hydration process of cyclohexene.

## 2. Materials and methods

### 2.1 Functionalization of zeolites with organosilanes

The HZSM-5 zeolite was obtained from Nankai University, China. We chose this HZSM-5 zeolite with 25 Si/Al ratio because it has a high acid site density to better promote cyclohexene hydration. (Octyl)-trimethoxysilane (OTS) was obtained from MACKLIN. Methylbenzene, ethyl alcohol and carbon tetrachloride were provided by SINOPHARM.

**Fig. 1:**
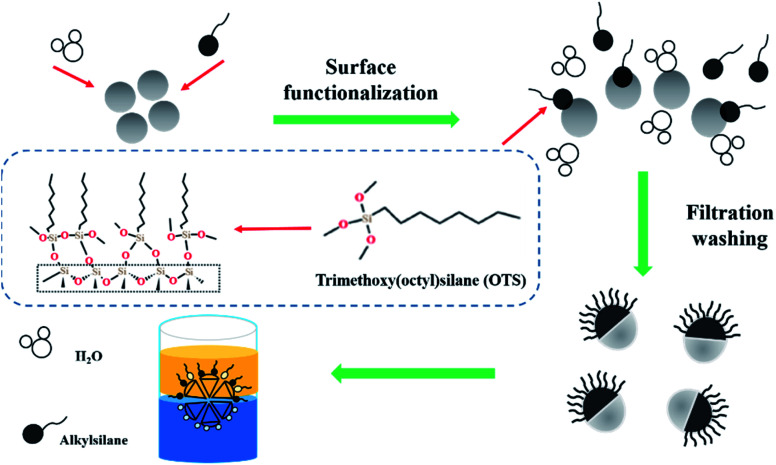
Hydrophobic functionalization of the HZSM-5 zeolite.

For the functionalization of the silanol groups, 1.65 g water was dispersed in 100 mL methylbenzene by sonication using a Horn sonicator (Fisher Scientific 600 W, 20 kHz) at 35% amplitude. It was then stirred by a magnetic stirrer (CJJ78-1) for 20 min. 5 g of the untreated HZSM-5 zeolites were added into the mixture and stirred for 30 min. Further, a 1.65 g solution of OTS was added in the suspension. The mixed liquor was stirred for 1 h at room temperature. Then, the zeolite was collected through filtration using a nylon filter (0.22 μm pore size), followed by washing with ethyl alcohol and carbon tetrachloride. The silylation of the zeolite was dried at 120 °C. This zeolite was named OTS(25%)-HZSM-5. The hydrophobic functionalization of the HZSM-5 zeolite is shown in Fig. 1.

Different loadings of (octyl)-trimethoxysilane (OTS) were prepared, but the amount of OTS and water were difference. For OTS(20%)-HZSM-5, 1.25 g solution of OTS and 1.25 g of water were chosen; for OTS(25%)-HZSM-5, 1.65 g solution of OTS and 1.65 g of water were chosen; for OTS(30%)-HZSM-5, 2.15 g solution of OTS and 2.15 g of water were chosen; for OTS(35%)-HZSM-5, 2.69 g solution of OTS and 2.69 g of water were chosen; for OTS(40%)-HZSM-5, 3.35 g solution of OTS and 3.35 g of water were chosen.
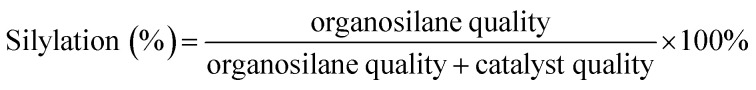


### 2.2 Study of cyclohexene hydration

The hydration of cyclohexene was performed in a 100 mL stainless-steel reactor on oil bath, to which water, catalysts and cyclohexene were sequentially added. The catalyst evaluations were carried out under different conditions. The reactants and products were analyzed *via* gas chromatography (7890B GC; Agilent Technologies, Santa Clara, CA, USA) and equipped with a mass spectrometer and flame ionization detector for product identification and quantification, respectively. The cyclohexene conversion was calculated as follows:
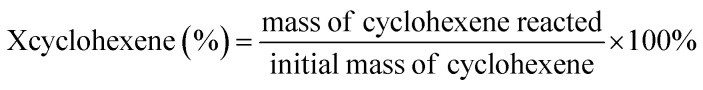


## 3. Results and discussion

We first confirmed the successful grafting of OTS onto the HZSM-5 zeolite and characterized the changes in the surface properties. These analyses were performed *via* Fourier-transform infrared spectroscopy (FTIR), contact angle (CA), N_2_-adsorption/desorption (BET), temperature-programmed desorption (TPD), pyridine-IR, X-ray photoelectron spectroscopy (XPS), thermal gravimetric analysis (TGA) and X-ray diffraction (XRD) techniques. Then, we evaluated the effects of OTS grafting onto the HZSM-5 zeolite in the cyclohexene hydration reaction under various conditions. According to the catalyst evaluation result, HZSM-5 and OTS(25%)-HZSM-5 were chosen for characterization measurements.

### 3.1 Characterization of the organosilane-grafted HZSM-5 zeolites

#### 3.1.1 Fourier-transform infrared spectroscopy (FTIR) analysis

**Fig. 2:**
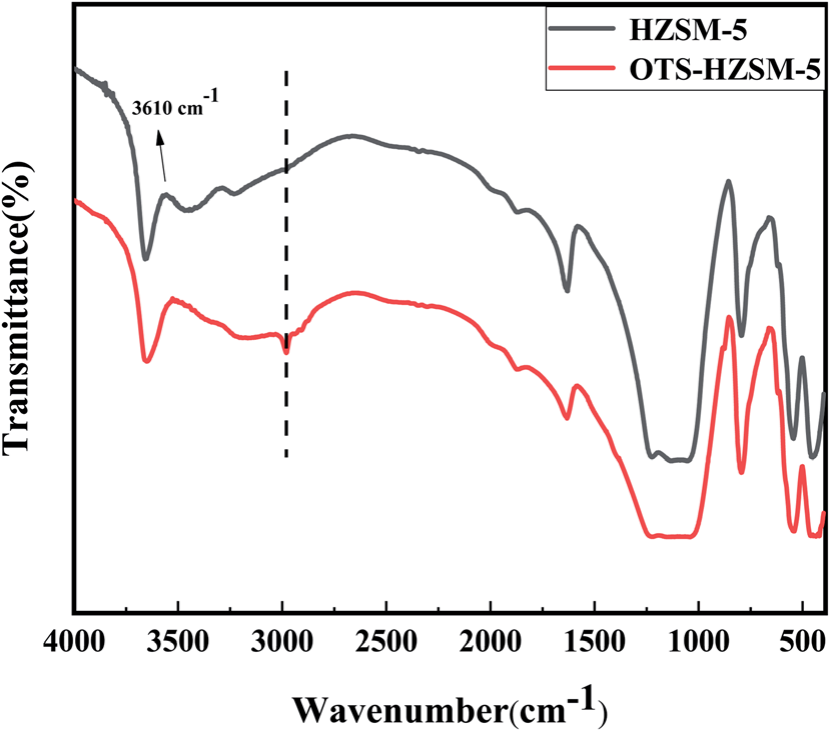
FT-IR spectra of HZSM-5 zeolite and OTS-HZSM-5 zeolite (Si/Al = 25).

The FTIR spectra confirmed the successful grafting of OTS onto the HZSM-5 zeolite. The skeletal FTIR spectra of the OTS-HZSM-5 zeolites presented an asymmetric stretching of the C–H bridges at 2800 and 3000 cm^−1^ (Fig. 2), which is attributed to the stretching vibrations of OTS.^27,28^ The breaking of the hydrogen bonds on the zeolite surface could form “Si–O–Si” bonds, which led to the C8 hydrophobic chain get adsorbed on the zeolite surface.^29^ Therefore, the new peaks indicated that a densely hydrophobic layer was formed on the zeolite surface. From Fig. 2, the Bronsted acid sites can be seen at 3610 cm^−1^, and this result is in agreement with information obtained in the study by Qin Zhengxing *et al.*^30^ Compared with the spectra of HZSM-5, the absorption intensity of hydroxyl groups decreased in OTS-HZSM-5, which indicated that surface wettability may have changed from hydrophilic to hydrophobic.

#### 3.1.2 Contact angle analysis

**Fig. 3:**
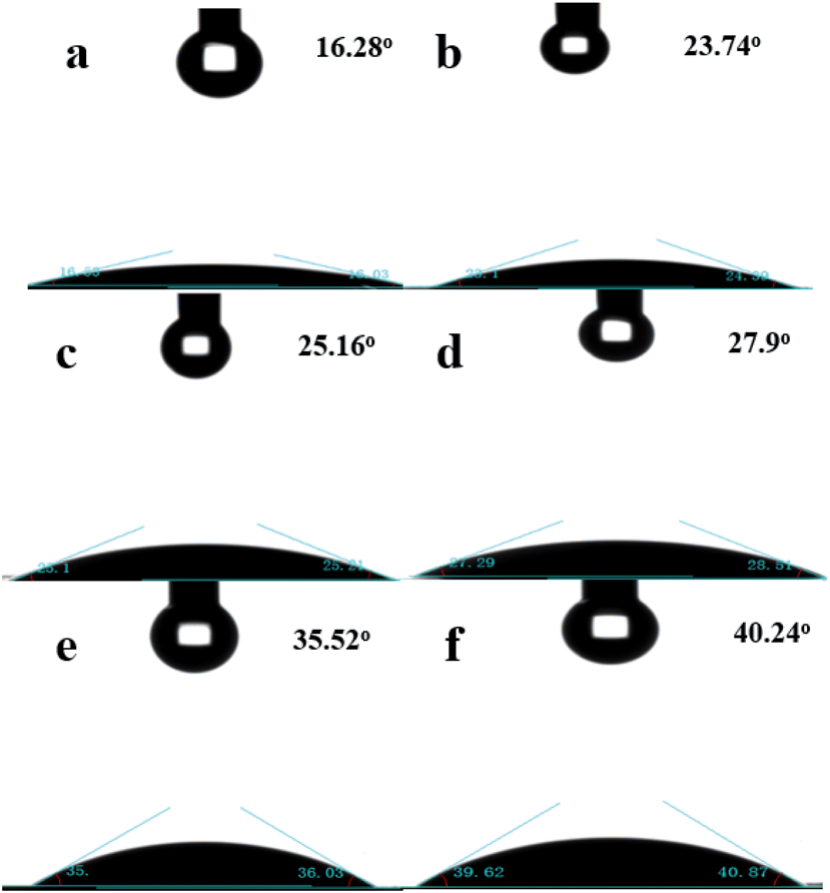
Contact angle variation on HZSM-5 zeolites at different amounts of OTS: (a) untreated HZSM-5, (b) OTS(20%)-HZSM-5, (c) OTS(25%)-HZSM-5, (d) OTS(30%)-HZSM-5, (e) OTS(35%)-HZSM-5, and (f) OTS(40%)-HZSM-5.

The contact angle (CA) was measured to determine the degree of hydrophobicity of the OTS-HZSM-5 zeolite. As shown in Fig. 3, the samples with the untreated HZSM-5 zeolite showed good hydrophilicity with a CA of 16.28°. The C8 hydrophobic chain could improve the hydrophobicity of the zeolite and lead the CA of OTS-HZSM-5 zeolites exceeded 20°. The CA results were different from the report, which the CA of the ZSM-5 zeolite was as high as ∼150° by Han *et al.*^27^ While preparing the catalyst, water was added to partly occupy the surface of the zeolite. The amphiphilic HZSM-5 was obtained by adjusting the ratio of water and OTS. Since the number of –O–(Si–CH_2_–)_2_–(CH_2_)_8_–CH_3_ groups was different, the CA was changed with the different amount of OTS.

#### 3.1.3 N_2_-adsorption/desorption analysis

**Fig. 4:**
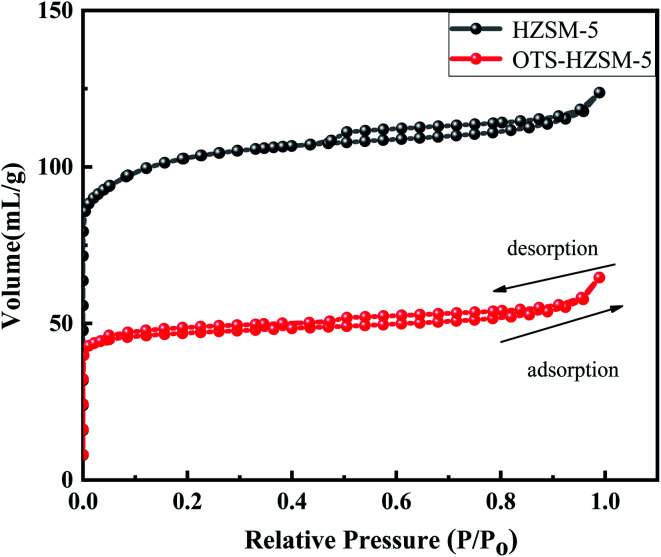
Adsorption isotherms for nitrogen at 77 K on the HZSM-5 zeolite and OTS-HZSM-5 zeolite (Si/Al = 25).

The N_2_ adsorption isotherms of the HZSM-5 and OTS-HZSM-5 zeolites and the calculated pore structure parameters are shown in Fig. 4 and Table 1, respectively. The isotherms of the HZSM-5 and OTS-HZSM-5 zeolites exhibited identical Langmuir type I isotherms.^31,32^ The OTS adsorption increased the resistance of the zeolite surface to N_2_ molecules, which made the hysteresis loop of OTS-HZSM-5 wider. The reason possibly was that the regularity of HZSM-5 was destroyed. Previous studies have reported that grafting organosilanes onto zeolites could reduce the surface area and pore volume as the pores were occupied by the organosilanes.^33^ After modifying with OTS, the BET surface area and the pore volume were reduced (the BET surface area changed from 311 to 144 m^2^ g^−1^, and the pore volume changed from 0.18 to 0.09 cm^3^ g^−1^). Since the kinetic diameter of OTS was larger than the ZSM-5 pore diameter (the intrinsic pore of ZSM-5 was 0.53 nm × 0.56 nm),^27,34^ OTS could not enter the pores, which the C8 hydrophobic chain was deposited on the outer surface of zeolite. The C8 hydrophobic chain deposited on the outer surface could improve the hydrophobicity of the zeolite. The silanol groups of the zeolite reacted with OTS to form “Si–O–Si” bonds, which could lead to the decline of the specific surface area and pore volume.

**Table 1:** Calculated composition of the HZSM-5 zeolite and OTS-HZSM-5 zeolite (Si/Al = 25)

Sample	BET surface area (m^2^ g^−1^)	Pore volume (cm^3^ g^−1^)		
		*V*_total_	*V*_micro_	*V*_meso_
HZSM-5	311	0.18	0.12	0.06
0TS-HZSM-5	144	0.09	0.07	0.02

#### 3.1.4 TPD and pyridine-IR analysis

The cyclohexene hydration reaction is an acid-catalyzed reaction; the acid sites of the porous structure played an important role in the catalytic performance.^35–37^ The density of the Brønsted and Lewis acid sites in the zeolites depend on the Si/Al ratio, the framework configuration, and the type of the cation used for charge compensation.^38^ Moreover, it could be considered that the functionalization occurred preferentially on the external surface of the zeolite and caused a significant loss in the acid density. To answer this question, the HZSM-5 and OTS-HZSM-5 zeolites were characterized *via* TPD and pyridine-IR.

**Fig. 5:**
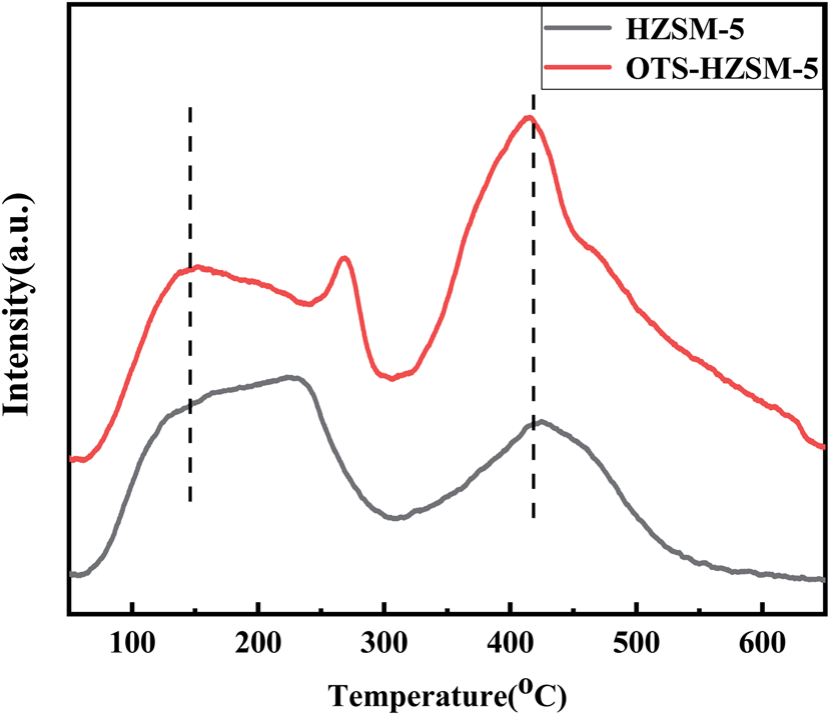
TPD profile of the HZSM-5 zeolite and OTS-HZSM-5 zeolite (Si/Al = 25).

In Fig. 5, NH_3_-TPD exhibited characteristic desorption peaks at 155 °C and 420 °C; the desorption peak at a lower temperature corresponded to the weak acid center and the desorption peak at a higher temperature corresponded to the strong acid center. The OTS-HZSM-5 zeolite had a clear protruding profile, which corresponded to the thermal decomposition of OTS at approximately 250 °C. It could be observed that the Brønsted and Lewis acid sites remained unchanged even after the modification.

**Fig. 6:**
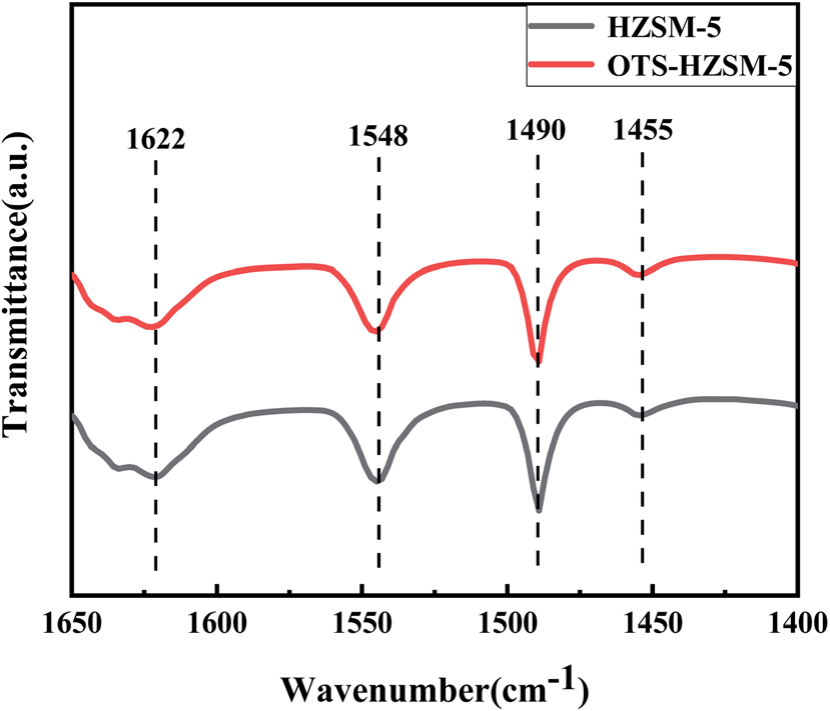
Pyridine-IR spectra of the HZSM-5 zeolite and OTS-HZSM-5 zeolite at 200 °C and atmospheric pressure.

In Fig. 6, the pyridine exhibited characteristic absorption bands at 1548 cm^−1^, 1455 cm^−1^, 1622 cm^−1^ and 1490 cm^−1^ after chemisorption on the acidic zeolites. These characteristic peaks corresponded to the Brønsted acid sites, the Lewis acid sites, and the central fractions of B and L acids. As shown in the spectra and Table 2, the peak intensity of OTS-HZSM-5 became weaker and the total acid content also decreased, which indicated that the Brønsted and Lewis acids slightly decreased after the OTS functionalization. It could be due to the fact that the C8 hydrophobic chain formed “Si–O–Si” bonds on the catalyst surface to cover part of the acidic sites.

**Table 2:** Calculated acid of the HZSM-5 zeolite and OTS-HZSM-5 zeolite (Si/Al = 25)

Sample	Acid area		Acid amount (mmol g^−1^)		Total acid (mmol g^−1^)
	Brønsted	Lewis	Brønsted	Lewis	
HZSM-5	8.69	1.64	352	88	441
0TS-HZSM-5	8.01	1.38	319	62	382

#### 3.1.5 XPS analysis

XPS is an important surface chemical analysis technique that could be utilized to analyze the surface property of a material.^27^ It could be observed from Table 3, and the effective coverage with organosilanes resulted in a strong increase in the carbon signal and a decrease in both the silicon and oxygen signal intensities.^39^ The XPS studies suggested that the OTS could form covalent bonds in the HZSM-5 zeolite surface, forming an organic coating layer. This result is in agreement with the information obtained from FTIR spectroscopy.

**Table 3:** Calculated composition of the HZSM-5 zeolite and OTS-HZSM-5 zeolite (Si/Al = 25)

Samples	Concentration of various elements (%)		
	C	O	Si
HZSM-5	43.86	38.24	17.9
OTS(25%)-HZSM-5	45.96	36.91	17.13

#### 3.1.6 TGA analysis

**Fig. 7:**
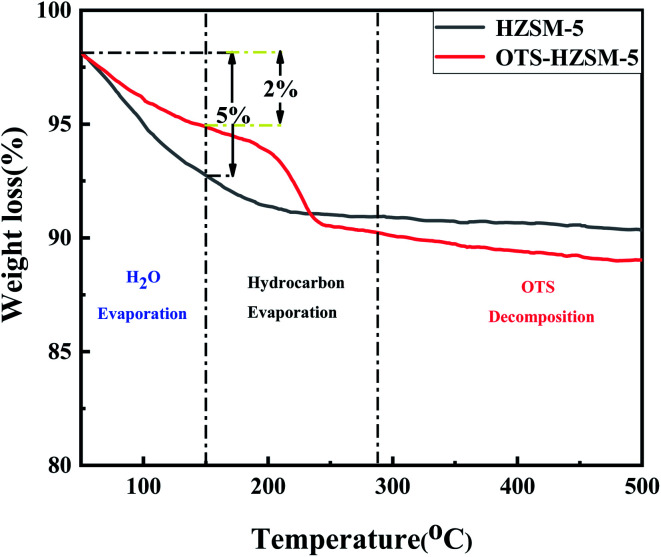
Thermogravimetric analysis (TGA) of the HZSM-5 zeolite and OTS-HZSM-5 zeolite (Si/Al = 25); left axis: weight (%).

TGA was used to assess the stability of OTS-HZSM-5 and quantify the amount of OTS grafted onto HZSM-5. As shown in Fig. 7, the weight loss of the zeolite occurred in three steps: (1) 150 °C, (2) 150–280 °C, and (3) 280–500 °C. The weight loss was the hydrolysis absorption and evaporation of residual organic solvents in the first stage. OTS-HZSM-5 (about 2%) was lower than HZSM-5 (about 5%), which was attributed to the fact that the HZSM-5 had more silanol groups.^40–42^ This result suggested that the OTS-HZSM-5 zeolite was more hydrophobic than the HZSM-5 zeolite. Compared with HZSM-5, the weight loss of OTS-HZSM-5 had a larger decline in the second and third stages, ad this was attributed to the slow decomposition of OTS at a higher temperature. These changes indicated that OTS grafted onto the HZSM-5 zeolite had better hydrophobicity.

#### 3.1.7 XRD analysis

**Fig. 8:**
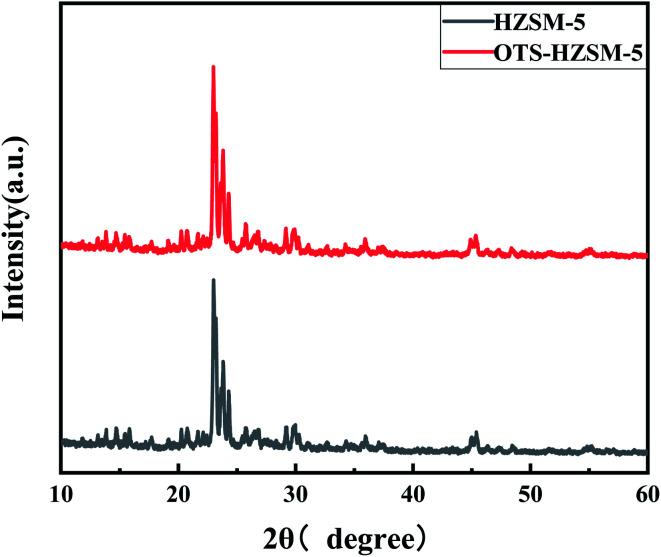
XRD pattern of the HZSM-5 zeolite and OTS-HZSM-5 zeolite (Si/Al = 25).

The XRD pattern of OTS-HZSM-5 confirmed its high crystallinity, as shown in Fig. 8. These results suggested that HZSM-5 and OTS-HZSM-5 had the same crystallinity, indicating that the functionalization of the silanol groups could not change the topology structure of the zeolite crystals.^43^

**Fig. 9:**
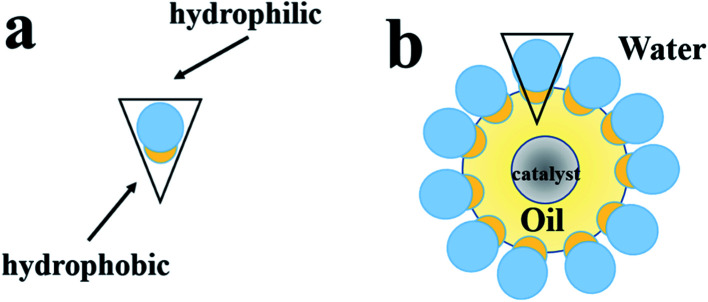
Droplet composition of the OTS-HZSM-5 zeolite (a and b).

### 3.2 Emulsion formation by modified catalysts in two-phase systems

Since the surface of the zeolite contained numerous free silanol groups, HZSM-5 was hydrophilic. It could not be suspended in the lower polar cyclohexene phase, and the modified catalyst could be suspended in the middle of the oil–water phase interface. Thus, the catalyst suspension behaviors were investigated by placing HZSM-5 and OTS-HZSM-5 in cyclohexene hydration systems.

**Fig. 10:**
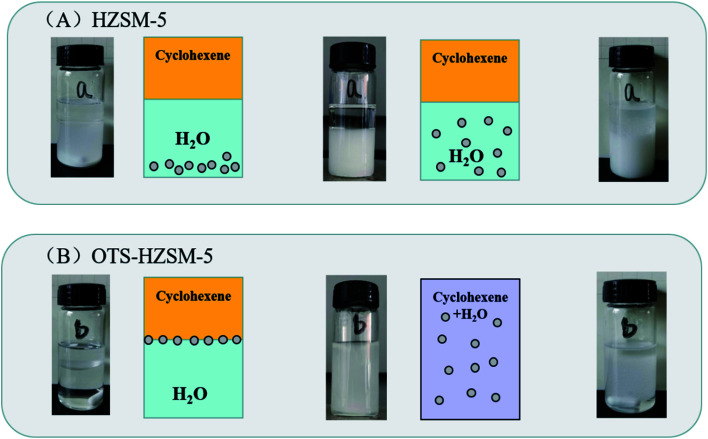
Suspension behaviors of the HZSM-5 and OTS-HZSM-5 zeolites in the cyclohexene system (A and B).

**Fig. 11:**
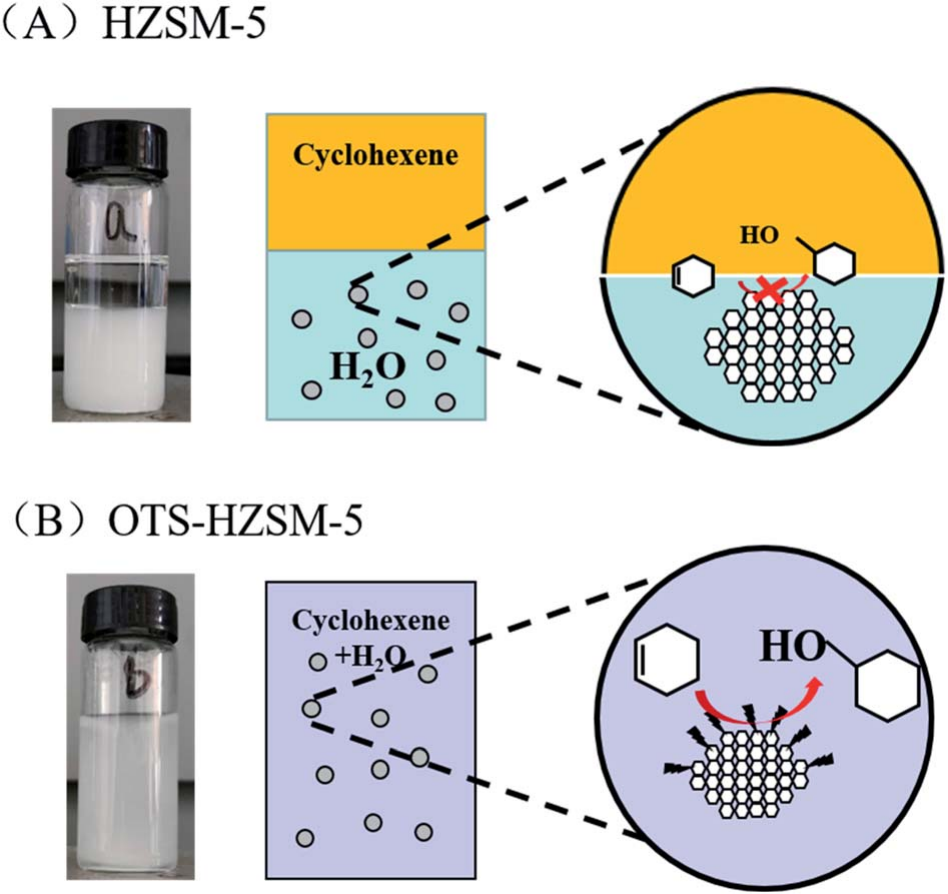
The cyclohexene hydration of the HZSM-5 zeolite and OTS-HZSM-5 zeolite (A and B).

Fig. 10A illustrates the suspension of the untreated HZSM-5 zeolite. Due to the hydrophilic nature of zeolite, the HZSM-5 zeolite was at the bottom of the water phase in the water–cyclohexene system. A single emulsion was formed by stirring, and the contact between the catalyst and reactants was only a layer of the phase interface, which led to the low conversion in the cyclohexene hydration reaction (Fig. 11A). When the hydrophobic catalyst was added, OTS-HZSM-5 was suspended in a two-phase interface (Fig. 10B). For the OTS-HZSM-5 zeolite, a uniform Pickering emulsion was formed by stirring, which could increase the contact area of the two phases to improve the conversion (Fig. 11B).

The droplet of the three-layer structure was formed by stirring. By magnifying the droplet, it was found that the outermost layer of the zeolite was hydrophilic, the secondary outer layer was oleophilic and the inner layer was a solid catalyst (Fig. 9a and b). The reaction activity was restricted due to the mass transfer limitations, which restricted the contact between the reactants. OTS-HZSM-5 could increase the contact between the two reactants, which formed a Pickering emulsion between the two immiscible reactants (Fig. 10B). Therefore, the limitation of the interfacial mass transfer was minimized, which increased the cyclohexene conversion. These visual observations were consistent with the assumption that OTS was grafted onto HZSM-5 to add a hydrophobic layer on the HZSM-5 surface.

### 3.3 Catalytic activity of OTS-HZSM-5 catalysts in cyclohexene hydration

**Fig. 12:**
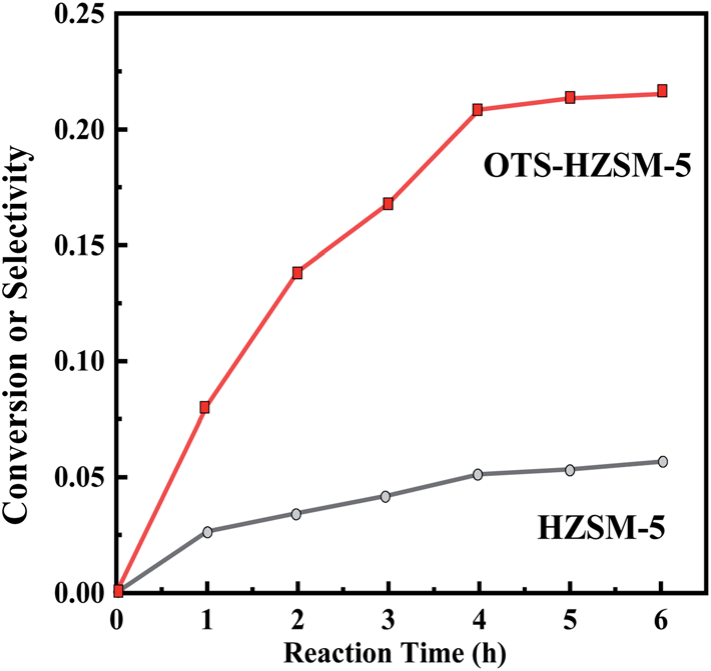
Cyclohexene conversion of the HZSM-5 zeolite and OTS-HZSM-5 zeolite in different reaction times.

Fig. 12 compares the cyclohexene conversion between two zeolites at different reaction times. A clear difference is observed: OTS-HZSM-5 had a good catalytic efficiency, and the cyclohexene conversion increased from 0 to 22.08% with reaction times. When the reaction time was increased from 0 to 6 h, HZSM-5 was only 5.18%.

The change of conversion was investigated under stirring and static environment in Table 4. Due to the fact that stirring could increase the contact area of the two reactants, the cyclohexene conversion significantly increased after stirring in the HZSM-5 zeolite. For the OTS-HZSM-5 zeolite, the cyclohexene conversion did not significantly improve. In the environment with a reaction temperature of 130 °C, both water and cyclohexene reached boiling points. The boiling phenomenon caused the three phases to form a uniform Pickering emulsion, which greatly increased the contact area in the reaction system. Due to the need of the industrialization of catalytic distillation, the catalyst was fixed in the tower. Considering the above-mentioned observations, we chose to evaluate the catalysts in the static state.

**Table 4:** Cyclohexene conversion of the HZSM-5 zeolite and OTS-HZSM-5 zeolite in stir and static environments

Type of catalyst	Stir conversion (%)	Static conversion (%)
HZSM-5	9.81	5.03
OTS-HZSM-5	21.13	20.87

To further compare the stability of these catalysts in the reaction process, the following experiments were performed. The reactants (water, catalyst and cyclohexene) were added to a reaction kettle. After the reaction, the product was cooled in a water bath. The oil phase and water phase were separated *via* centrifugation, and then chromatographic analysis was performed with the quantitative addition of the internal standard ethanol. The separated solid catalyst was washed with ethyl alcohol and carbon tetrachloride, and dried in an oven overnight at 120 °C. To maintain the hydrophobicity of the OTS-HZSM-5 zeolite, no other high-temperature regeneration methods were used. Since a significant fraction of the catalyst was lost during the separation, washing, and drying processes, we repeated multiple sets of experiments and recovered them to evaluate the zeolite life experiment.

**Fig. 13:**
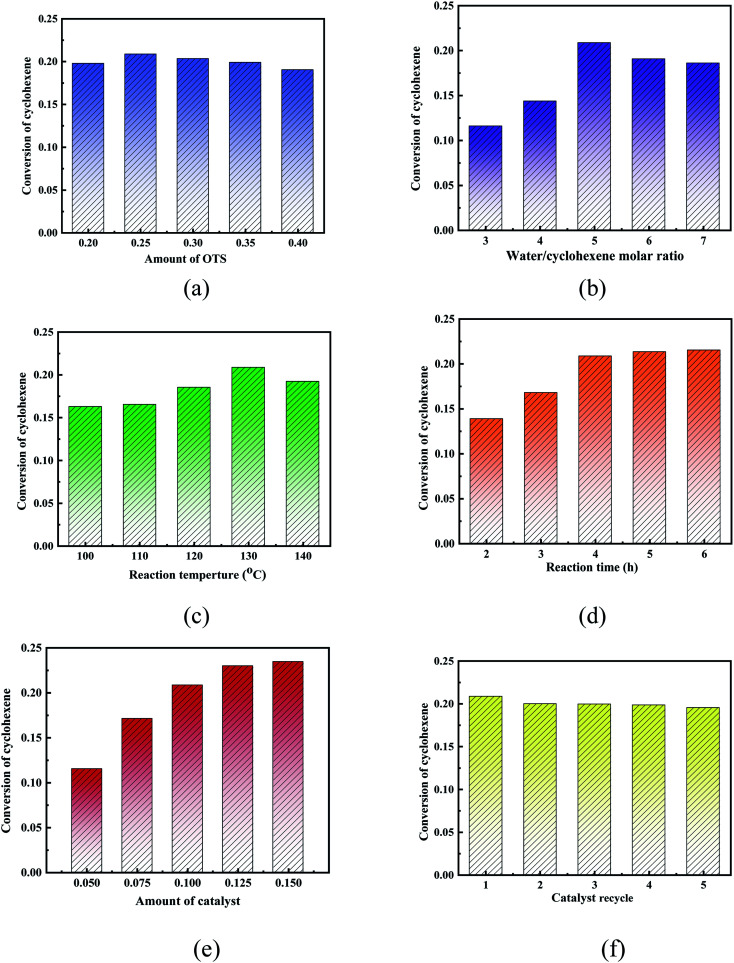
Cyclohexene conversion of the HZSM-5 zeolite and OTS-HZSM-5 zeolite (Si/Al = 25). (a) The different functionalization (20%, 25%, 30%, 35%, and 40%); (b) the different water–olefin molar ratio (3, 4, 5, 6, and 7); (c) the different reaction temperature (110 °C, 120 °C, 130 °C, 140 °C, and 150 °C); (d) the different reaction time (2 h, 3 h, 4 h, 5 h, and 6 h); (e) the different amount of catalyst (5%, 7.5%, 10%, 12.5%, and 15%); (f) catalyst recycle (1, 2, 3, 4, and 5). Optimum reaction conditions: OTS(25%); water/cyclohexene molar ratios = 5; reaction temperature = 130 °C; reaction time = 4 h; catalyst dosage = 10%.

The OTS-HZSM-5 zeolites with different functionalization (20%, 25%, 30%, 35%, and 40%) were prepared by adjusting the ratio of water and (octyl)-trimethoxysilane (OTS). When the degree of silylation was 25%, the conversion was as high as 20.87%. When the degree of silylation further increased, the conversion decreased. Since the OTS could form “Si–O–Si” bonds with the surface of the HZSM-5 zeolite, the C8 hydrophobic chain could block a part of the pores and inactivate a part of the acidic sites. As the amount of the OTS reagent increased, the C8 hydrophobic chain could wrap the surface of the catalyst and reduce the specific surface area, thus decreasing cyclohexene conversion. So, the OTS(25%)-HZSM-5 was chosen (Fig. 13a).

The cyclohexene hydration reaction was a three-phase coexistence reaction, different water–olefin molar ratios had different conversion, which proper molar ratios could promote the hydration reaction. The cyclohexene conversion was evaluated when the molar ratios of water–cyclohexene were 3, 4, 5, 6 and 7, respectively. It observed that the conversion first increased and then decreased. Excessive water could promote the reaction of carbocation with water molecules to produce protonated alcohols, which increased conversion. When water was added after the reaction reached equilibrium, the conversion could not increase significantly. So, the water–cyclohexene molar ratios were 5 (Fig. 13b).

The reaction temperature was investigated at 100 °C, 110 °C, 120 °C, 130 °C, 140 °C and 150 °C in the cyclohexene hydration reaction. As the reaction temperature increased, the collision of molecules increased to improve the reactant contact. Furthermore, since cyclohexene hydration is a reversible exothermic reaction, when the reaction temperature was too high, the reaction proceeded in the reverse direction. So, the reaction temperature was considered as 140 °C (Fig. 13c).

The cyclohexene conversion showed an increasing trend and then stabilized with an increase in the reaction time. As the reaction time increased, the reaction slowly achieved equilibrium. So, the reaction time was considered as 4 h (Fig. 13d).

The amount the catalyst was investigated with mass percentages of 5%, 7.5%, 10%, 12.5% and 15% (relative to the quality of water). Increasing the amount of the catalyst could improve the dispersion in the aqueous and oil phases and reduce the interfacial mass transfer limitations. When the amount of the catalyst was too large, the cyclohexene conversion remained constant. This was because the reaction achieved the state of equilibrium. To ensure cost effectiveness, the catalyst mass fraction was considered as 10% (Fig. 13e).

In this group of experiments, the stability of the catalyst was studied by recycling the catalyst. Simultaneously, the OTS-HZSM-5 zeolite retained a large fraction of activity (∼90%) after regeneration. It was found that the color of the catalyst gradually changed from initial white to dark yellow as the number of the catalyst cycles increased. As the catalyst was used repeatedly, the polar products were adsorbed on the catalyst pores, which blocked the catalyst pores and gradually reduced the catalyst activity (Fig. 13f).

## 4. Conclusion

The cyclohexene hydration reaction was an acid-catalyzed reaction, and the functionalization of the silanol groups could improve hydrophobicity, which could not cause a significant loss in the acid density. The OTS-HZSM-5 zeolite was synthesized, which was confirmed by FT-IR, CA and BET characterizations. The catalytic performance was conducted in the cyclohexene hydration, and the results showed that the OTS-HZSM-5 zeolite exhibited better stability and conversion (20.87%). Compared with the unmodified HZSM-5, which was due to the OTS-HZSM-5 could form an uniform Pickering emulsion to increase the two phases contact area. Hydrophobic zeolites were promising for other acid-catalyzed reactions to enhance industrial applications.

## Conflicts of interest

There are no conflicts to declare.

## Supplementary Material
